# A Diverse Repertoire of Human Immunoglobulin Variable Genes in a Chicken B Cell Line is Generated by Both Gene Conversion and Somatic Hypermutation

**DOI:** 10.3389/fimmu.2015.00126

**Published:** 2015-03-20

**Authors:** Philip A. Leighton, Benjamin Schusser, Henry Yi, Jacob Glanville, William Harriman

**Affiliations:** ^1^Crystal Bioscience Inc, Emeryville, CA, USA; ^2^Department of Veterinary Science, Institute for Animal Physiology, Ludwig-Maximilians-Universitaet Muenchen, Munich, Germany; ^3^Distributed Bio Inc, San Francisco, CA, USA

**Keywords:** gene conversion, somatic hypermutation, DT40, antibody repertoire, chicken B cells, human antibodies, deep sequencing

## Abstract

Chicken immune responses to human proteins are often more robust than rodent responses because of the phylogenetic relationship between the different species. For discovery of a diverse panel of unique therapeutic antibody candidates, chickens therefore represent an attractive host for human-derived targets. Recent advances in monoclonal antibody technology, specifically new methods for the molecular cloning of antibody genes directly from primary B cells, has ushered in a new era of generating monoclonal antibodies from non-traditional host animals that were previously inaccessible through hybridoma technology. However, such monoclonals still require post-discovery humanization in order to be developed as therapeutics. To obviate the need for humanization, a modified strain of chickens could be engineered to express a human-sequence immunoglobulin variable region repertoire. Here, human variable genes introduced into the chicken immunoglobulin loci through gene targeting were evaluated for their ability to be recognized and diversified by the native chicken recombination machinery that is present in the B-lineage cell line DT40. After expansion in culture the DT40 population accumulated genetic mutants that were detected via deep sequencing. Bioinformatic analysis revealed that the human targeted constructs are performing as expected in the cell culture system, and provide a measure of confidence that they will be functional in transgenic animals.

## Introduction

Historically, therapeutic monoclonal antibodies have been derived from immunized mice and phage display technologies. However, antigens that are conserved throughout mammalian evolution are typically weakly or non-antigenic in mice. In some cases, the failure to elicit an immune response in mice has been obviated by immunizing chickens (1–3). Early attempts to use chicken-derived antibodies were thwarted by the lack of technology to derive monoclonal antibodies from non-murine animals. A fusion partner for chicken B cells was identified to create an avian version of the classical murine hybridoma technology (4) although it has not gained wide usage and phage display has been used more frequently to isolate chicken monoclonals (5–11). We developed technology to isolate antigen-specific monoclonal antibodies from immunized chickens using a single lymphocyte screening and recovery method, the gel-encapsulated microenvironment (GEM) assay (see US Patents 8030095 and 841517382). The GEM assay involves placing a single antibody-secreting lymphocyte in proximity with reporters (which can be cells or beads). The secreted antibody diffuses locally within the GEM and has the opportunity to bind to the reporters. Bound antibody can be detected either directly through the use of a secondary antibody or by eliciting a response in the reporter that generates a visual signal. Each GEM may contain multiple types of reporters which can be differentiated from each other based on color. Selected GEMs are isolated and antibody genes are amplified through RT PCR and cloned into a mammalian expression vector, usually in scFv format.

The advantage of producing antibodies to conserved epitopes in chickens prompted the development of humanization protocols to obviate the immune response in patients to the avian V regions of chimeric antibodies (7, 8). An alternative approach to eliminating the anti-animal response in patients is to engineer the animal to produce human immunoglobulins (12). We are currently creating a line of chickens that will produce antibodies with fully human V regions. Human V regions will be recovered from these birds using GEMs. We will then combine the human V segments with human constant regions to produce fully human antibodies with therapeutic potential. The human V region sequences have been designed to replace the equivalent chicken coding regions while leaving most of the endogenous IgH and IgL regulatory sequences intact.

Diversification of chicken immunoglobulin genes is achieved through gene conversion (GC) and somatic hypermutation (SHM) (13). In humans, diversification is achieved through V(D)J recombination and SHM. Because of the phylogenetic distance between humans and chickens and the known differences in the mechanism of diversity generation, it was prudent to evaluate the genetically modified V regions *in vitro* before investing in the much longer timeline to produce genetically modified birds.

A preliminary evaluation of expression and diversification of human immunoglobulin V regions in DT40 cells was previously reported (14). Briefly, chicken V_L_ and V_H_ loci were knocked out in DT40 and replaced with human V_K_ (V_K_3-15) and V_H_ (V_H_3-23) genes. To achieve GC of human genes in chicken B cells, human pseudogene arrays were inserted upstream of the functional human V_K_ and V_H_ regions. The sequences of the V_K_ and V_H_ functional genes served as the starting template for the design of the human pseudogenes. Proper expression of chimeric IgM comprises human variable regions and chicken constant regions were shown. Sanger-based sequencing of selected DT40 genetic variants confirmed that the human pseudogene arrays contributed to the generation of diversity through GC at both the *Igl* and *Igh* loci. Although these data showed that engineered pseudogene arrays contribute to human antibody sequences in chicken B cells, a more thorough repertoire analysis was not possible as only a relatively small number of events were analyzed.

Here, we have used next-generation sequencing methods to study much more comprehensively the repertoire generated by a long-term, non-selected culture of DT40 cells harboring targeted human V genes, analyzing well over 1 million sequences for each of the heavy and light chains. We are now able to show that the engineered locus can produce a diverse pool of human antibody sequences in chicken B cells.

## Materials and Methods

### Culture of chicken DT40 cells carrying human V genes

**Figure 1:**
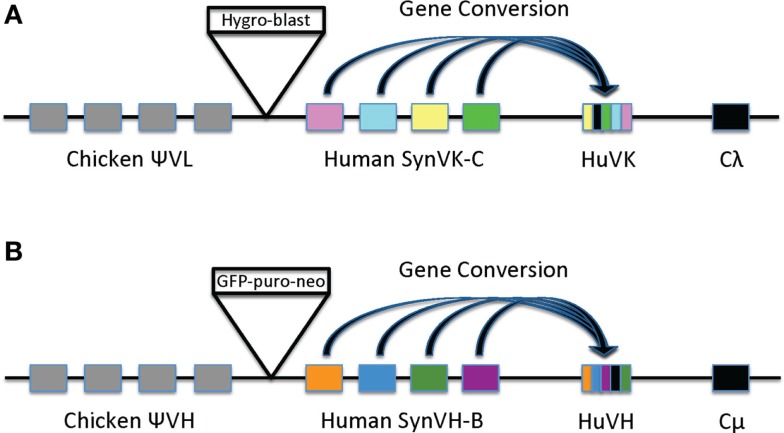
**Diagrams of light chain (A) and heavy chain (B) loci in cell line 1208-1**. **(A)** In the light chain, the endogenous rearranged chicken V_L_ and its promoter in DT40 was replaced by an array of human SynVK-C pseudogenes and a rearranged functional HuV_K_ gene driven by the chicken V_L_ promoter. The chicken Ψ V_L_ pseudogene array, constant region (Cλ), J–C intron, and 3′ flanking DNA are intact. A β-actin-hygromycin, β-actin- blasticidin resistance cassette (box labeled Hygro-blast) was placed between the chicken and human pseudogene arrays as part of the transfection process. **(B)** In the heavy chain, the endogenous rearranged chicken V_H_ and 350 bp of its promoter region were replaced by the SynVH-B human pseudogene array, the chicken V_H_ promoter, and a rearranged functional human V_H_ gene. The upstream chicken Ψ V_H_ pseudogene array, the chicken JH-Cμ intron, and constant regions are intact. A β-actin-EGFP, β-actin-puromycin, β-actin-neomycin selectable marker cassette (box labeled GFP-puro-neo) was placed between the chicken and human pseudogene arrays as part of the transfection process. Gene conversion in both heavy and light chains is depicted as blocks of sequences (colored blocks) being transferred from the pseudogenes to the HuV_K_ and HuV_H_ functional genes.

**Figure 2:**
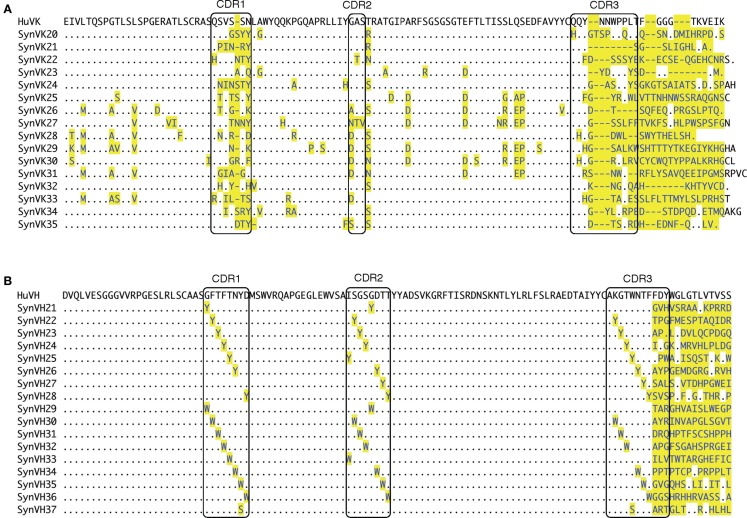
**Human light and heavy chain pseudogene sequences**. **(A)** Alignment of SynVK pseudogenes in the SynVK-C construct. Top line shows the sequence of the functional HuV_K_ gene that is mutated by the SynVK pseudogenes. The CDRs (boxes; IMGT nomenclature) were derived from human EST databases. Some pseudogenes also contain framework changes derived from the ESTs. At the 3′ end of the pseudogenes, the sequence of the flanking DNA downstream of CDR3 in each pseudogene is shown. This flanking sequence is part of the 100 bp spacer sequence inserted between each pair of pseudogenes. **(B)** Alignment of the SynVH pseudogenes in the SynVH-B construct. Top line shows the sequence of the functional HuV_H_ gene. The CDRs consist of a tyrosine/tryptophan/serine scan. The framework regions contain no changes.

A derivative of the chicken B cell line DT40 was made in which the chicken immunoglobulin variable regions were replaced with human variable regions in both the IgL and IgH loci (14). In both loci, the active functional allele was targeted, thereby switching the cells from expressing normal chicken surface IgM to the expression of chimeric IgM, consisting of human variable regions and chicken constant regions. A derivative of DT40, cell line 1208-1, was produced by serial transfection with knockout constructs followed by site-specific insertion of constructs for the expression of human V regions. To take advantage of the GC machinery in DT40, upstream arrays of human-sequence pseudogenes were included in the transgenes to provide the donor sequences for mutating the single functional human kappa (HuV_K_) and human heavy chain (HuV_H_) regions (Figure 1). Pseudogene arrays were synthesized by Bio Basic (Markham, ON, Canada). These pseudogenes were based on the sequences of the functional HuV_K_ and HuV_H_ regions, with diversity incorporated into the complementarity determining regions (CDR), and in some cases, the framework regions as well (Figure 2). The pseudogenes were thus designed *de novo* and not based on the endogenous pseudogenes found in the human genomic heavy and light chain loci. We refer to the HuV_K_ pseudogenes as the SynVK array and the HuV_H_ pseudogenes as the SynVH array. Diversity in the SynVK array was derived from human EST sequences, whereas the SynVH array was made by scanning substitution of CDR positions with tyrosine, tryptophan, or serine residues. Furthermore, additional AID hotspots (nucleotides WRC/GYW) were incorporated into the SynVK-C construct, as silent changes. In the 1208-1 cell line, construct SynVH-B was inserted at the heavy chain locus, followed by insertion of the SynVK-C construct at the light chain locus. The sequences of the pseudogene arrays are shown in Figure 2.

The 1208-1 cell line was propagated for 10 weeks with both SynVK-C and SynVH-B transgenes to allow mutations to accumulate prior to harvesting genomic DNA for sequencing (additionally, the precursor cell line carrying only the SynVH-B construct was cultured for 3 weeks before transfection of the SynVK-C construct). The culture was expanded to 1.85e8 cells and gDNA was purified by Qiagen DNeasy kit.

### Generation of amplicons for sequencing

Purified gDNA from the 1208-1 DT40 cell line was sent for further processing to Genewiz, Inc. The HuV_K_ and HuV_H_ regions were amplified using the primers in Table 1. Amplicons were sequenced by Genewiz, Inc. (South Plainfield, NJ, USA) on the Illumina MiSeq 2x250 platform (Illumina, Inc., San Diego, CA, USA). Raw data files are available online at the NCBI sequence read archive (SRA), project PRJNA275158, accession number SRP055184.

**Table 1:** **Primers for amplification of HuV_K_ and HuV_H_ sequences from gDNA from 1208-1 cells**.

For HuV_H_ amplicon	Sequence
HuVHampf1 (forward)	TCCTTCCCCACAGGTGTC
HuVHampr1 (reverse)	GGTTGAAGACTCACCTGAG
**For HuV_K_ amplicon**	
HuVKampf1 (forward)	CAGACTGCACCGGAGAAA
HuVKampr1 (reverse)	GTCAGCGACTCACGTTTG

### Sequence data analysis

High throughput sequencing reads were analyzed using VDJFasta (http://www.distributedbio.com/vdjfastadocs/), a general antibody repertoire algorithm suitable for interpretation of engineered antibody diversity. In order to control for possible residual chicken content, we used a combination natural chicken and human synthetic segment classification database. In order to analyze the repertoire comprehensively in a manner unbiased by the underlying GC mechanics, we used general profile Hidden–Markov models to identify immunoglobulin content and align sequences in a consistent manner independent of nucleotide composition. Kabat positional annotations were transferred from Hidden–Markov model columns to every aligned sequence in the resulting database, enabling consistent annotation of frameworks and CDR boundaries (15–17).

## Results

### Read quality analysis

Analysis was performed by processing all reads through VDJFasta. Sequences were assigned closest segments with a probabilistic classifier. All reads were translated into six-frames of translation and analyzed for Ig content by profile-Hidden–Markov model scoring with VDJFasta, with a 1e-10 cutoff for significance. Pass-cutoff frames were aligned using the pHMMs and analyzed for framestate and coverage. Over 96% of reads contained full-length clones, with over 1 million HuV_H_ reads and 2 million HuV_K_ reads available for downstream analysis (Table 2).

**Table 2:** **Analysis of on-target proportions in data**.

	HuV_H_		HuV_K_	
	Reads	Proportion (%)	Reads	Proportion (%)
Total	1117578	100.0	2066629	100.0
wV	1115812	99.8	2029766	98.2
wJ	1093284	97.8	2014189	97.5
wVJ	1093270	97.8	2014173	97.5
wVJclone	1074927	96.2	2002661	96.9

*wV, contains detectable V-segment; wJ, contains detectable J-segment; wVJ, contains both a detectable V and J segment; wVJclone, contains a detectable V and J segment, and a CDR3 that remains in-frame*.

### Comparison to inserted arrays of V genes

Nucleotide and translated CDRs were extracted from profile Hidden–Markov model alignments, using minimum profile annealing cutoffs to ensure high fidelity CDR capture [see Ref. (15)]. CDRs were compared to a reference database containing all V genes and pseudogenes that were included in the targeted array. Counts of exact match to reference database were stored for all CDRs.

### Sequence complexity

The extracted sequences were highly redundant in both the heavy chain and light chain data sets, with the single functional human V gene being seen predominately in its respective group. The non-mutated V_K_ represented 81% of all the full-length sequence reads and the non-mutated V_H_ 57% of the total. These non-mutated sequences are referred to as the “reference” sequences (one for V_H_ and one for V_K_). For the heavy chain, 9125 unique clones were found at a minimum 2× sequence depth; for the light chain, 7671 unique clones were found. If the sequences are counted at a 1× sequence depth a total of 21,403 unique heavy chains and 33,848 unique light chain genes were seen.

### Identifying gene conversion and somatic hypermutation events

Each framework and CDR was analyzed separately, with an exact-match assignment performed to reference synthetic human frameworks designed into the transgenic organism. A control search was also performed with all known native IgL and IgH chicken segments, but they were never encountered in the repertoire. GC events were scored if a sequence found in the V_K_ (Table 3) or V_H_ (Table 4) pool could be traced back to particular pseudogenes present in the array. In the cases where gene converted sequences are shared by multiple pseudogenes, one GC event is counted and all possible donor pseudogenes are indicated. Individual GC events were counted at the 1× sequence depth since it is unlikely that stretches of nucleotides would occur through sequencing or read error. Regardless, all CDR events classified as GC occurred more than twice in the data set. Within each CDR a high proportion of the unique sequences matched perfectly with the reference sequence. Those that deviated from the reference sequence in ways that could not be clearly attributed to GC are labeled as “SHM or fusion” and this category includes single or multiple point mutations as well as possible complex events (i.e., multiple sequential GC). Sequencing errors would be expected to show up in this category, possibly inflating the observed events.

**Table 3:** **Number of unique SHM and gene conversion sequences within CDRs for light chain**.

CDR1	Events	CDR2	Events	CDR3	Events
SynVK_ Reference	28735	SynVK_ Reference	17179	SynVK_ Reference	13995
SHM or fusion	3853	SHM or fusion	15340	SHM or fusion	22630
SynVK20	20	SynVK20/21	160	SynVK23_ CDR3	87
SynVK21	44	SynVK22	76	Chicken pseudo	0
SynVK22	39	SynVK24	21		
SynVK23	219	SynVK26	83		
SynVK24	195	SynVK27	11		
SynVK25	121	SynVK28/29	203		
SynVK26	52	SynVK30/31	284		
SynVK27	44	SynVK32	204		
SynVK28	30	SynVK33	129		
SynVK29	49	SynVK34	88		
SynVK30	314	SynVK35	70		
SynVK31	39	Chicken pseudo	0		
SynVK32	4				
SynVK33	15				
SynVK34	27				
SynVK35	48				
Chicken pseudo	0				
Sum GC	1260	Sum GC	1329	Sum GC	87
Total	33848	Total	33848	Total	36712^a^

*^a^Includes a small proportion of non-full-length reads*.

**Table 4:** **Number of unique SHM and gene conversion sequences within CDRs for heavy chain**.

CDR1	Events	CDR2	Events	CDR3	Events
SynVH_ Reference	19440	SynVH_ Reference	5600	SynVH_ Reference	19379
SHM or fusion	647	SHM or fusion	15096	SHM or fusion	1830
SynVH_21	185	SynVH_21	111	SynVH_22	13
SynVH_22	120	SynVH_22	61	SynVH_23	11
SynVH_23	39	SynVH_23	138	SynVH_24	32
SynVH_24	160	SynVH_24	3	SynVH_25	21
SynVH_25	96	SynVH_25	15	SynVH_26	20
SynVH_26	39	SynVH_26	149	SynVH_27	9
SynVH_28	79	SynVH_27	22	SynVH_28	3
SynVH_29	38	SynVH_28	28	SynVH_30	4
SynVH_30	37	SynVH_29	66	SynVH_31	20
SynVH_31	102	SynVH_30	12	SynVH_32	18
SynVH_32	101	SynVH_31	19	SynVH_37	43
SynVH_33	17	SynVH_32	4	Chicken pseudo	0
SynVH_34	116	SynVH_33	5		
SynVH_35	5	SynVH_34	48		
SynVH_36	169	SynVH_35	11		
SynVH_37	13	SynVH_36	15		
Chicken pseudo	0	Chicken pseudo	0		
Sum GC	1316	Sum GC	707	Sum GC	194
Total	21403	Total	21403	Total	21403

### Evaluation of potential contribution of endogenous chicken pseudogenes

The IgL and IgH knockouts were made by deleting portions of the functional VJC and VDJ regions, respectively, by homologous recombination. Since endogenous chicken pseudogenes remain upstream of the inserted human V gene array, they could in principle contribute to repertoire diversity. We specifically checked for such events by creating a library of all known chicken pseudogenes and running the analysis as we did with the library containing our human pseudogenes. Evidence of endogenous pseudogenes participating in GC events was never observed.

### Gene conversion and SHM in framework regions

Since some diversity was incorporated into the frameworks of the inserted V_K_ pseudogenes, it was possible to identify GC events as well as SHM events in these regions (Table 5). Fewer GC attributable sequences were found in the V_K_ frameworks as compared to the V_K_ CDRs; however, this may be due simply to the lower framework diversity that was incorporated in the pseudogene design.

**Table 5:** **Number of unique SHM and gene conversion sequences within V_K_ framework regions**.

FW1	Events	FW2	Events	FW3	Events
SynVK_ Reference	26576	SynVK_ Reference	17114	SynVK_ Reference	22447
SHM or fusion	7163	SHM or fusion	16405	SHM or fusion	11235
SynVK22	43	SynVK24	94	SynVK23	144
SynVK25	41	SynVK27	32	SynVK25	11
SynVK26	13	SynVK28	54	SynVK27	10
SynVK27	8	SynVK29	24	SynVK30	1
SynVK30	1	SynVK33	49		
SynVK31	1	SynVK34	76		
SynVK33	2				
Sum GC	109	Sum GC	329	Sum GC	166
Total	33848	Total	33848	Total	33848

### Identification of multiple gene conversion events

In some cases, we were able to identify sequences with contributions from two different pseudogenes, and these are termed paired-fusion events (Tables 6–8). Partial GC was analyzed using the parsimonious assumption of single-conversion events within the CDR as a source of non-100% identity match to the SynVH pseudogene segment reference database. Custom software was written to generate all non-redundant fusion events that can emerge between pairwise interactions of the reference database sequences. Paired-fusion events are highly biased in their relative occurrence, as an analysis of the most commonly encountered rearrangements demonstrates. Paired-fusion events were not feasible to determine for SynV_K_ due to the sequence complexity inherent in this array.

**Table 6:** **Top 10 most common HuV_H_ CDR-H1 paired-fusion events**.

**FREQ**	
67	SynVH_22_CDR_1 invades > SynVH_21_CDR_1 at position 2
21	SynVH_25_CDR_1 invades > SynVH_21_CDR_1 at position 13
17	SynVH_21_CDR_1 invades > SynVH_28_CDR_1 at position 3
13	SynVH_22_CDR_1 invades > SynVH_29_CDR_1 at position 1
12	SynVH_27ref_CDR_1 invades > SynVH_36_CDR_1 at position 22
11	SynVH_21_CDR_1 invades > SynVH_27ref_CDR_1 at position 1
10	SynVH_25_CDR_1 invades > SynVH_32_CDR_1 at position 11
8	SynVH_23_CDR_1 invades > SynVH_21_CDR_1 at position 7
7	SynVH_27ref_CDR_1 invades > SynVH_37_CDR_1 at position 20
6	SynVH_36_CDR_1 invades > SynVH_21_CDR_1 at position 23

**Table 7:** **Top 10 most common HuV_H_ CDR-H2 paired-fusion events**.

**FREQ**	
4304	SynVH_26_CDR_2 invades > SynVH_29_CDR_2 at position 13
1381	SynVH_21_CDR_2 invades > SynVH_32_CDR_2 at position 10
562	SynVH_26_CDR_2 invades > SynVH_29_CDR_2 at position 13
318	SynVH_21_CDR_2 invades > SynVH_30_CDR_2 at position 4
185	SynVH_28_CDR_2 invades > SynVH_27_CDR_2 at position 19
115	SynVH_21_CDR_2 invades > SynVH_32_CDR_2 at position 10
80	SynVH_21_CDR_2 invades > SynVH_31_CDR_2 at position 7
78	SynVH_23_CDR_2 invades > SynVH_29_CDR_2 at position 13
49	SynVH_22_CDR_2 invades > SynVH_29_CDR_2 at position 13
41	SynVH_CDR_2 invades > SynVH_21_CDR_2 at position 23

**Table 8:** **Top 10 most common HuV_H_ CDR-H3 paired-fusion events**.

**FREQ**	
204	SynVH_21_CDR_3 invades > SynVH_35_CDR_3 at position 20
153	SynVH_21_CDR_3 invades > SynVH_24_CDR_3 at position 10
39	SynVH_21_CDR_3 invades > SynVH_36_CDR_3 at position 23
30	SynVH_21_CDR_3 invades > SynVH_27_CDR_3 at position 19
21	SynVH_37_CDR_3 invades > SynVH_21_CDR_3 at position 14
13	SynVH_27_CDR_3 invades > SynVH_21_CDR_3 at position 19
8	SynVH_24_CDR_3 invades > SynVH_21_CDR_3 at position 10
7	SynVH_36_CDR_3 invades > SynVH_21_CDR_3 at position 23
7	SynVH_22_CDR_3 invades > SynVH_21_CDR_3 at position 4
6	SynVH_25_CDR_3 invades > SynVH_21_CDR_3 at position 14

### Positional variation profiling of the repertoire

**Figure 3:**
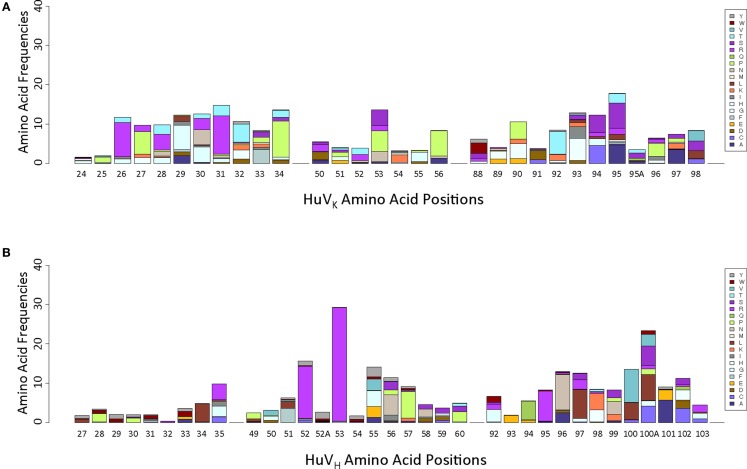
**Positional weight matrices of non-reference residue variation, by Kabat position**. **(A)** HuV_K_ variation, indicated positionally. **(B)** HuV_H_ variation, indicated positionally.

Analysis of positional amino acid variation was performed by converting a total alignment of non-redundant amino acid sequences into a positional weight matrix (PWM), with reference residue frequency omitted to emphasize non-reference residue variation (Figure 3). The number of amino acids observed at each position cannot be attributed to GC events including both complete replacements and single paired fusions. These observations suggest that the diversity generated by GC is augmented by SHM.

## Discussion

The DT40 cell line has been used extensively to better understand the nature of immunoglobulin diversification in chickens, including the mechanism of GC (18–21). The cell line has also been used to develop chicken antibodies to novel targets using *in vitro* selection strategies (22, 23). We have inserted human V gene arrays into the chicken immunoglobulin loci of DT40. In principle, the human V genes in DT40 cells could be diversified *in vitro* to provide an unselected library of immunoglobulin sequences from which antigen-specific antibodies could be extracted. However, most therapeutic antibodies are derived from immunized animals producing affinity-matured, antigen-specific antibodies. In the current context, we have used DT40 cells to provide *in vitro* proof of concept that arrays of human-derived immunoglobulin gene sequences can be diversified by chicken B cells. Subsequently, these sequences will be introduced into chickens to provide genetically engineered animals that can be immunized to produce affinity-matured, antigen-specific antibodies with therapeutic potential. Thus, our purpose with DT40 is to determine whether targeted synthetic human V gene arrays can be used as a substrate for genetic diversification in chicken cells in a way that mirrors what is known regarding the native chicken immunoglobulin loci. Affirmative data in the DT40 culture system inspires confidence that the effort required on the arduous path to generating a genetically engineered chicken will be rewarded with a transgenic animal that performs as expected.

We have previously shown that our heavy and light chain arrays can be diversified by both GC and SHM in DT40 cells (14). This analysis involved conventional sequencing of a few hundred clones sampled from a large population of DT40 cells. Some examples of expected diversification events were seen, but most events were likely missed at that depth. Next generation sequence technology allows for identification of very rare events in a non-selected, non-biased cellular population. Indeed, we were able to show in the current work that every pseudogene in our array was used by some cell in the population. The finding of paired-fusion events, wherein GC occurs using two different pseudogenes in succession is expected in a fully functional locus. It has been estimated that wild-type chicken B cells undergo 1–2 independent GC events on average during affinity maturation (24).

We were also able to confirm our previous conclusion that for both heavy and light chains, GC is more prevalent in CDR1 and CDR2 than CDR3, which is heavily skewed toward SHM. This finding is also consistent with previously published results (25). Nonetheless, a mix of both templated and non-templated mutations is seen in all CDRs, resulting in a repertoire with amino acid diversity at every CDR position. It will be interesting to see if a similar bias can be seen in the SynV chicken, in which processes of cellular selection may affect the repertoire in a way that is not seen in DT40, which has no selection pressure for surface Ig expression or specificity.

While it is reasonable to use deep sequencing on our DT40 population to determine whether certain types of events have occurred, caution should be used in interpreting the observed frequency results because of the nature of the long-term DT40 culture system. In such a system, clones with particular sequences could have a growth advantage, or alternatively, a particular mutation could occur very early in the expansion of the culture and then subsequent mutations could occur in addition, potentially creating a large number of “unique clones,” which carry the original mutation. For instance, in our V_H_CDR2 data, we find a very large number of clones bearing a S53R substitution (Figure 3). The high frequency observed could be the result of a true mutational hotspot that mutated many times independently, or simply the result of a single random mutation that occurred early in the expansion of the population. One sequence bearing this substitution is highly redundant in our sample, second only to the starting sequence; this is consistent with the existence of a large subpopulation wherein secondary mutations could have occurred. Further, if S53R is an aberration, it skewed our data to make it appear that SHM in HC CDR2 is extremely high relative to GC, which may not be the correct interpretation.

In summary, the DT40 culture system, coupled with deep sequencing methodologies, is an excellent tool for the functional testing of arrays of synthetic human V genes designed to be diversified and affinity-matured *in vivo*. Our deep sequencing results confirm that arrays of human V genes can be targeted into the immunoglobulin loci of chicken cells and the host machinery can diversify those genes over time in a manner that recapitulates *in vivo* GC in the B cells of wild-type chickens. Furthermore, when rare events are included in the analysis, it is clear that even in a relatively small population of cells, all of the introduced pseudogenes are capable of participating in GC, and that codons for non-templated amino acid residues are generated through SHM in every CDR of both light chain and heavy chain. These data support the concept of introducing constructs containing all necessary genetic elements required for diversification in the B cell compartment, contributing to a functionally diverse repertoire of human-sequence antibodies in a transgenic chicken. Once made, this bird will be the most evolutionarily divergent host of any human-Ig transgenic animal currently available, and will be particularly well suited to generating novel antibodies to therapeutic targets that are conserved among mammals.

## Conflict of Interest Statement

Crystal Bioscience has an issued patent (US 8592644) regarding the production of human antibodies in transgenic chickens.
